# Genomic microsatellites identify shared Jewish ancestry intermediate between Middle Eastern and European populations

**DOI:** 10.1186/1471-2156-10-80

**Published:** 2009-12-08

**Authors:** Naama M Kopelman, Lewi Stone, Chaolong Wang, Dov Gefel, Marcus W Feldman, Jossi Hillel, Noah A Rosenberg

**Affiliations:** 1 Porter School of Environmental Studies, Department of Zoology, Tel Aviv University, Ramat Aviv, Israel; 2 Center for Computational Medicine and Bioinformatics, University of Michigan, Ann Arbor, Michigan, USA; 3 Department of Medicine, Barzilai Hospital, Ashkelon, Israel; 4 Department of Biology, Stanford University, Stanford, California, USA; 5 Robert H Smith Institute of Plant Sciences and Genetics, Faculty of Agriculture, The Hebrew University of Jerusalem, Rehovot, Israel; 6 Department of Human Genetics, University of Michigan, Ann Arbor, Michigan, USA; 7 Life Sciences Institute, University of Michigan, Ann Arbor, Michigan, USA

## Abstract

**Background:**

Genetic studies have often produced conflicting results on the question of whether distant Jewish populations in different geographic locations share greater genetic similarity to each other or instead, to nearby non-Jewish populations. We perform a genome-wide population-genetic study of Jewish populations, analyzing 678 autosomal microsatellite loci in 78 individuals from four Jewish groups together with similar data on 321 individuals from 12 non-Jewish Middle Eastern and European populations.

**Results:**

We find that the Jewish populations show a high level of genetic similarity to each other, clustering together in several types of analysis of population structure. Further, Bayesian clustering, neighbor-joining trees, and multidimensional scaling place the Jewish populations as intermediate between the non-Jewish Middle Eastern and European populations.

**Conclusion:**

These results support the view that the Jewish populations largely share a common Middle Eastern ancestry and that over their history they have undergone varying degrees of admixture with non-Jewish populations of European descent.

## Background

Large-scale genomic studies have contributed to a growing body of knowledge about the population structure of a wide variety of human populations [1-5]. Such studies have enabled precise inferences about the relationships of closely related groups, about the extent to which individuals in neighboring populations can be genetically distinguished, and about the potential of genetics for inference of ancestry at the intracontinental level. In general, Jewish populations, whose genetic origins and population relationships have long been of interest, have been excluded from such studies or examined only peripherally. Although some studies have included members of Jewish populations in the context of analyses of broader geographic regions [6-9], Jewish populations have only recently become a focus of investigation for genome-wide studies of population structure [10].

The population genetics of Jewish populations has been considered primarily from the perspective of the Y chromosome and mitochondrial DNA, and in smaller-scale studies using as many as 20-30 autosomal genetic markers. Although several studies have supported a genetic affinity among most Jewish populations, potentially due to shared ancestry [11-16], others have suggested similarity between Jewish and non-Jewish populations as a result of some level of gene flow among groups [12,14,17-19]. The discovery of shared Y chromosomes common in separate Jewish populations from different geographic regions has strengthened the evidence for shared Jewish genetic ancestry, but as evidenced in the considerable attention given in Israel to the 2008 scholarly book "When and how was the Jewish people invented" [20], debate continues regarding the issue of whether separate Jewish populations have any deep shared genetic ancestry beyond that shared with non-Jewish groups. The difficulty of fine-scale resolution of Jewish population relationships is highlighted by the different conclusions reached in two early genetic investigations that proceeded concurrently using similar data on classical markers, and that even today remain among the most comprehensive evaluations of Jewish population relationships [13,17]. Whereas Karlin *et al*. [13] observed that most Jewish populations had lower genetic distance to other Jewish populations than to non-Jewish European and Middle Eastern populations included in their study, Carmelli & Cavalli-Sforza [17] found that a discriminant analysis scattered Jewish populations among clusters corresponding to various non-Jewish European and Middle Eastern groups.

Increasing the number of autosomal markers used in population-genetic studies has the potential to provide more detailed information that may help to resolve the population structure of Jewish populations and their historical neighbors. Here we extend the use of genome-wide markers to evaluate genetic relationships among Jewish populations and other Middle Eastern and European populations. To assess patterns of genetic structure among Jewish populations as well as the relationship of Jewish genetic variation to that of other populations, we examine 678 microsatellites in a collection of 78 individuals of Jewish descent representing four groups defined by community of origin, as well as genotypes of 321 Middle Eastern and European non-Jewish individuals at the same markers. We find that the Jewish populations cluster together in several analyses, separately from the remaining populations. In addition, we find that the genetic ancestry of the Jewish populations is intermediate such that in several types of analysis of population structure, the Jewish populations are placed centrally, between the Middle Eastern populations and the European populations. These results are compatible with an ancient Middle Eastern origin for Jewish populations, together with gene flow from European and other groups in the Jewish diaspora.

## Methods

### Samples

To compare the genetic variability of Jewish populations with that of other Middle Eastern and European groups, we examined a sample of 399 individuals, representing four Jewish groups defined by their origin prior to 20th century migrations, as well as 12 other Middle Eastern and European populations from the HGDP-CEPH Human Genome Diversity Cell Line Panel [21]. Our primary interest was in the relationship of Jewish populations to each other and to non-Jewish Middle Eastern and European populations. Previous analysis had demonstrated that the Middle Eastern and European HGDP-CEPH populations form genetic clusters separate from other populations such as those from Central and South Asia [4,22]. Because inclusion in population structure analyses of distant populations has the potential to obscure genetic differences that might exist among closely related populations [22,23], we did not include HGDP-CEPH populations from Central/South Asia or other geographic regions unlikely to be relevant for the genetic study of the Jewish populations analyzed.

The Middle Eastern populations included in the study were Bedouin (46), Druze (42), Mozabite (29), and Palestinian (46). The European populations were Adygei (17), Basque (24), French (28), Italian (13), Orcadian (15), Russian (25), Sardinian (28), and Tuscan (8). Middle Eastern and European non-Jewish individuals were taken from the H952 subset of the HGDP-CEPH panel [24]. The Jewish samples included Ashkenazi Jews (20), Moroccan Jews (20), Tunisian Jews (20), and Turkish Jews (20). Two Tunisian Jewish individuals were omitted from the analysis following a procedure for detection of relatives (see below). Jewish individuals were sampled at the Barzilai Medical Center in Ashkelon, Israel, and included immigrants and second-generation immigrants from the source populations. Informed consent was obtained from all participants, and the project was approved by the ethics committee of the Barzilai Medical Center.

### Markers

The Jewish individuals were genotyped by the Mammalian Genotyping Service for microsatellite loci in Marshfield Screening Sets 16 and 54 http://research.marshfieldclinic.org/genetics. The collection of markers genotyped in the Jewish populations overlaps to a large extent with a set of 783 markers previously reported for the HGDP-CEPH individuals [25,26], but is not completely identical to the earlier marker set. Thus, to enable comparison of the 80 newly included Jewish individuals with commensurable genotypes previously reported for the HGDP-CEPH individuals, data analysis was restricted to 678 loci typed across all populations. Preparation of genotypes for the Jewish populations proceeded in the same manner as the preparation of genotypes in the study of Wang *et al*. [27], which used the same set of 678 markers; for the Middle Eastern and European non-Jewish populations, the data used here are the same as in that study, except that we considered only individuals from the H952 subset that excluded close relatives.

### Detection of relatives

Considering all pairs among the 80 Jewish individuals, we examined identity-by-state sharing to detect relatives. In addition, separately for each Jewish population we screened pairs of individuals for close relatives by utilizing the *RELPAIR *program [28,29]. Both approaches were applied in a similar manner to that used in a previous study [24]. Two second-degree relative pairs were detected in the Tunisian sample, and for each pair, one individual was omitted from further analysis (individuals 2345 and 2348).

### Genetic diversity

Expected heterozygosity was computed by using the sample-size-corrected estimator, averaging across loci to obtain an overall estimate [30]. Paired values for individual loci were used in Wilcoxon signed-rank tests of heterozygosity across populations. For each locus, the number of distinct alleles and the number of private alleles, that is, alleles unique to one population, were measured as functions of the number of sampled chromosomes. This analysis used the rarefaction procedure, as implemented in *ADZE *[31], averaging the number of distinct alleles and the number of private alleles across possible subsets of sampled chromosomes while adjusting for differences in sample size across populations. We obtained the mean number of distinct alleles and the mean number of private alleles for each of three combined sets of samples (European, Jewish, Middle Eastern), averaging across loci. Our *ADZE *analysis used only 656 of the 678 loci, omitting loci with >15% missing data in any one of the three combined samples. This choice accords with that of Szpiech *et al*. [31], producing similar results to those obtained with all 678 loci while permitting higher numbers of sampled chromosomes to be considered.

### Jewish, Middle Eastern, and European population structure

The program *Structure 2.2.3 *[32] was used to assess population structure for the full dataset used in this study, using the *F *model of correlation in allele frequencies. The program *Structure *is the most widely used in a family of programs that cluster individuals based on their diploid genotypes, in an unsupervised manner, without using prior knowledge of their populations of origin (additional programs in this collection include *BAPS *[33,34], *mStruct *[35], and *Structurama *[36]). Using the admixture model of individual affiliations, for each individual *Structure *determines the fractions of genetic affiliation of the individual in each of a predetermined number of clusters (*K*). The admixture model is particularly suitable in complex populations for which mixed membership of individuals in multiple clusters is expected [32,37]. We ran *Structure *for *K *ranging from 2 to 16, with 40 replicates for each *K *and a burn-in period of length 30,000 iterations followed by 30,000 additional iterations. For each *K*, and for each pair of replicates, we determined the similarity of the estimated affiliations using the symmetric similarity coefficient (SSC) scores based on the best alignment of the replicates. This alignment was obtained using the LargeKGreedy algorithm of the software *CLUMPP *[38], with 10,000 random input sequences. Using a threshold of 0.8 for the SSC scores, we separated different convergence modes among the 40 replicates with a given value of *K*, where a mode was defined as a clique such that all pairs of replicates within the clique had SSC≥0.8. For each mode and each *K*, *CLUMPP *was again used to obtain the average cluster memberships of the replicates placed into the mode. The program *Distruct *[39] was used to produce plots of these average memberships. Our combined application of *Structure *and *CLUMPP *to summarize clustering results follows the approach employed in previous studies [2,27].

Multimodality in clustering solutions was observed for some values of *K*. The mode containing the largest number of replicates (the "major mode") for *K *= 2 contained 39 of 40 *Structure *runs. For *K *= 3 and *K *= 5, only one mode was found, containing all 40 runs. For *K *= 4, the major mode contained 15 of 40 runs. The second-largest mode contained 11 runs, and was very similar to the major mode of *K *= 5, except that it did not separate the Mozabites and the Bedouins (results not shown). For *K *= 6, the major mode contained 20 of 40 runs. The second-largest mode, containing 14 runs, was very similar to the major mode, except that it showed greater similarity of the Bedouins to the Palestinians (results not shown). For *K *= 7 and *K *= 8, the number of replicates in the major mode was well below half of the total number of replicates examined, equaling 13 for *K *= 7 and 12 for *K *= 8. Two new clusters were identified in the major mode for *K *= 8 compared to the analysis for *K *= 6; one of these clusters largely corresponded to the Tunisian Jews and the other largely corresponded to the Sardinians (results not shown). The major mode for larger values of *K *contained fewer replicates, at most 7 for values of *K*>8. For the larger values of *K *(*K*>8), the second-largest mode contained nearly as many replicates as the major mode - for example, for *K *= 7, *K *= 8, *K *= 9, and *K *= 10, the second-largest mode possessed 8, 7, 6, and 4 runs, respectively, compared to 13, 12, 7, and 5 replicates for the major mode. Because inferences based on *K*>6 were less replicable than those based on smaller values of *K*, we chose for display the major mode for each *K *from 2 to 6.

### Genetic distance and population trees

Neighbor-joining population trees were produced using the *neighbor *program in the software package *Phylip 3.65 *[40], considering each of three genetic distance measures. Distance measures were chosen among those found to produce relatively high bootstrap support in comparisons of multiple trees in past microsatellite studies [41-43]. The distance matrices for the allele-sharing distance (computed as one minus the proportion of shared alleles under Hardy-Weinberg proportions [44]), chord distance [45] and Nei's standard distance (computed as one minus Nei's identity [46]) were obtained with the software *Microsat *[47], bootstrapping across loci 10,000 times. For each collection of 10,000 bootstrap replicates, we constructed a majority-rule consensus tree, resolving multifurcations by sequentially incorporating the groupings that had the highest frequencies in the set of bootstraps and that were compatible with groupings already incorporated.

### Combinations of pairs of populations and their similarity to Jewish populations

For each Jewish population we examined the genetic distances between the allele frequency vector of that population and linear combinations of allele frequency vectors for pairs of other populations. For each Jewish population and each linear combination of two other populations, we obtained a mean allele-sharing genetic distance across loci. For each pair of populations considered in obtaining linear combinations, we examined combinations in which the fraction from the first population ranged from 0 to 1, with a step size of 0.01.

### Multidimensional scaling

Pairwise distances between individuals were calculated using allele-sharing distance [44]. We then performed multidimensional scaling (MDS) for the individual distance matrices using the *cmdscale *function in R. This function performs classical MDS based on the approach of Cailliez [48]. MDS analysis was also performed for several subsets of the full collection of individuals: Jewish individuals alone, Jewish and European individuals, Jewish and Middle Eastern individuals, and Jewish and Palestinian individuals.

In the two-dimensional MDS plots, we evaluated distances between groups of individuals by using the average linkage distance [49,50]. For a pair of groups in an MDS plot, this quantity, denoted here by *L*_0_, is the mean Euclidean distance between the location in the plot of a randomly chosen member of the first group and a randomly chosen member of the second group. The significance of the separation of two groups was evaluated by permutation of labels within groups, as specified in the contexts of the various plots. The probability that a random permutation of the labels gives rise to a smaller average linkage distance for two groups than that seen using the actual labels was obtained from a distribution of the average linkage distance across 1000 permutations. While the magnitude of a value of *L*_0 _is not itself meaningful, the relative size of *L*_0 _values for multiple pairs of groups in the same MDS plot carries information about the relative levels of separation of the various pairs.

### Jewish population structure

We also performed *Structure *analysis for the Jewish individuals alone. Using the same *Structure *model and the same lengths for runs as in the analysis of the full data, we considered values of *K *ranging from 2 to 6, performing 40 replicates for each value. *CLUMPP *and *Distruct *were used to process the *Structure *results in the same manner as in the analysis with the full dataset. We found that for *K *= 2, the major mode contained all 40 replicates, and that for *K*>2, the additional subdivision observed beyond that seen for *K *= 2 was negligible (results not shown).

## Results and Discussion

### Genetic variability

The mean heterozygosity across loci was compared among the 16 populations. Heterozygosity in human populations is generally predicted by proximity to Africa [25,51], so that European populations generally have lower heterozygosity values than Middle Eastern populations. The Jewish populations showed intermediate levels of heterozygosity within the range of values obtained for the European and Middle Eastern populations (Table 1). Among the Jewish populations, heterozygosity was slightly lower in the Tunisian Jewish population (*P *= 0.0063 for Tunisian vs. Ashkenazi, *P *= 1.77 × 10^-5 ^for Tunisian vs. Turkish, *P *= 0.169 for Tunisian vs. Moroccan, two-tailed Wilcoxon signed-rank tests). Combining the Jewish samples together, the mean heterozygosity of 0.734 across loci was slightly less than the corresponding value of 0.739 for the combined Middle Eastern samples (*P *= 0.0044, two-tailed Wilcoxon signed-rank test) and slightly greater than the value of 0.732 for the combined European samples (*P *= 0.0602, two-tailed Wilcoxon signed-rank test).

**Table 1:** Heterozygosity and sample size for European, Jewish, and Middle Eastern populations.

Population	Group	Number of individuals	Mean heterozygosity across loci	Standard deviation of heterozygosity across loci
Mozabite	Middle Eastern	29	0.739	0.082

Bedouin	Middle Eastern	46	0.736	0.077

Druze	Middle Eastern	42	0.723	0.086

Palestinian	Middle Eastern	46	0.734	0.080

Tunisian	Jewish	18	0.724	0.095

Moroccan	Jewish	20	0.730	0.088

Turkish	Jewish	20	0.736	0.083

Ashkenazi	Jewish	20	0.733	0.087

Sardinian	European	28	0.723	0.088

Italian	European	13	0.728	0.095

Tuscan	European	8	0.736	0.108

French	European	28	0.731	0.083

Basque	European	24	0.719	0.096

Orcadian	European	15	0.726	0.095

Russian	European	25	0.732	0.085

Adygei	European	17	0.730	0.088

**Figure 1:**
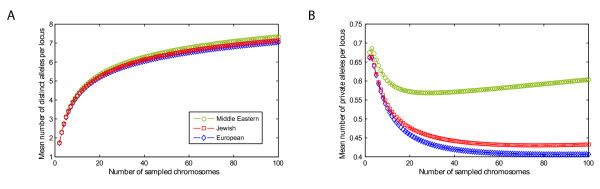
**Variability statistics as functions of the number of sampled chromosomes, for combined samples from European, Jewish, and Middle Eastern populations**. (a) The mean number of distinct alleles per locus. (b) The mean number of private alleles per locus.

The mean number of distinct alleles per locus and the mean number of private alleles per locus provide additional measures of genetic variability. Correcting for differences in sample size among the three groups (European, Jewish, Middle Eastern), the Jewish populations were intermediate in their number of distinct alleles per locus (Figure 1A). In addition, the mean number of private alleles per locus was smaller for the Jewish populations than for the Middle Eastern populations, but slightly greater than for the European populations (Figure 1B). Considering the list of values for all sample sizes investigated, the smaller values of the mean number of distinct alleles and private alleles for Jewish populations compared to Middle Eastern populations and the larger values for Jewish populations compared to European populations were statistically significant (*P *< 10^-17 ^for all comparisons, two-tailed Wilcoxon signed-rank tests).

### Population structure

**Figure 2:**
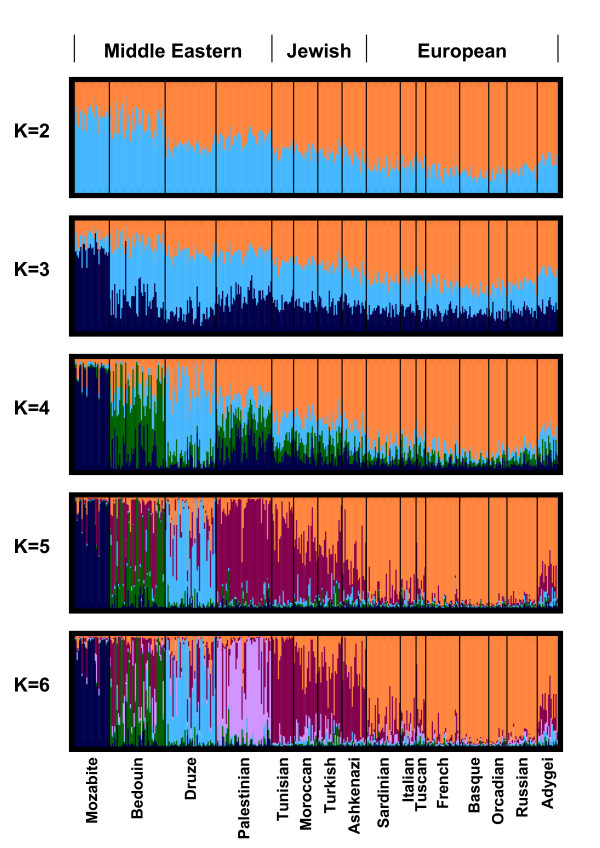
**Population structure for European, Jewish, and Middle Eastern populations, inferred with unsupervised clustering**. The number of predefined clusters (*K*) is indicated to the left of each plot. Each individual is represented by a thin vertical line that is partitioned into *K *colored components according to the inferred membership in *K *genetic clusters. For each *K*, only the major mode is shown (obtained in 39 of 40 replicates for *K *= 2, 40 of 40 replicates for *K *= 3, 15 of 40 replicates for *K *= 4, 40 of 40 replicates for *K *= 5, and 20 of 40 replicates for *K *= 6).

To study the similarities among the European, Jewish, and Middle Eastern populations, we used unsupervised model-based clustering as implemented in the *Structure *software package [32]. Figure 2 illustrates the major clustering solutions for each value of *K *from 2 to 6. For *K *= 2, the estimated population structure assigns the Jewish populations mixed ancestry in the two clusters, one of which has higher membership in Middle Eastern populations and the other of which has higher membership in European populations. Among the Middle Eastern populations, the Bedouins cluster closely with the Mozabites, a north African group from Algeria, while the Palestinians and Druze are placed closer to the Jewish and European populations. For *K *= 3, the Mozabite population largely separates from the other populations. For *K *= 4, the Druze, Bedouins and Palestinians are each largely distinct in cluster membership coefficients; the Jewish populations show somewhat greater similarity to these three Middle Eastern groups than do the European populations other than the Adygei, but they also have greater similarity to the European populations than do the Middle Eastern groups. Among the European populations, the Adygei population, from the Caucasus region, shows some similarity in cluster membership coefficients to the Jewish populations, especially to the Ashkenazi population (this similarity is also observable for *K *= 2 and *K *= 3). For *K *= 5, the new cluster produced contains most Palestinian individuals, as well as sizable components of the four Jewish populations, the Adygei and the Bedouins. For *K *= 6, this cluster is further subdivided, producing one cluster that corresponds mainly to Palestinians and one cluster that corresponds mainly to the Jewish populations and to a lesser extent, the Adygei and Bedouins.

**Figure 3:**
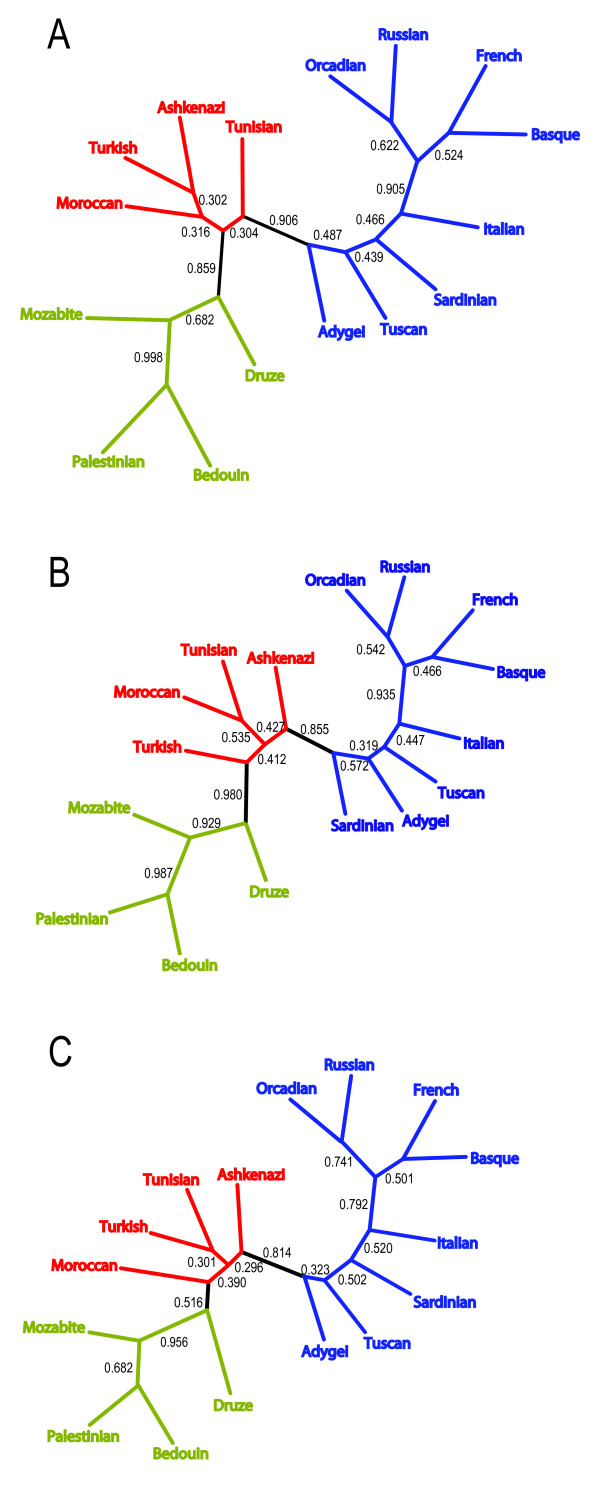
**Neighbor-joining population trees for European, Jewish, and Middle Eastern populations**. (a) Neighbor-joining tree based on the allele-sharing genetic distance. (b) Neighbor-joining tree based on the chord genetic distance. (c) Neighbor-joining tree based on Nei's standard genetic distance. External branches were colored to indicate the groups to which populations belong (blue - European; red - Jewish; green - Middle Eastern). Sequentially, internal branches were then colored if all colored branches to which they connected had the same color. The two remaining branches in black separate the European, Jewish, and Middle Eastern groups. The number on an edge represents the fraction of bootstrap replicates supporting that edge, among 10,000.

Neighbor-joining population trees obtained for the three distance matrices were generally quite similar (Figure 3). All three trees are divided into a European side and a Middle Eastern side, with the four Jewish populations located in the interior. This division is supported by relatively strong bootstrap values for the chord and allele-sharing distances (>0.85), and by somewhat lower bootstrap values for Nei's genetic distance (0.814 and 0.516). Two of the three trees identify the Ashkenazi population as the closest Jewish population to the European section of the tree, although bootstrap values for the associated branch are low; two of the three trees identify the Adygei population as the closest European population to the Jewish populations (also with low bootstrap values). The Middle Eastern section of the tree has the same structure for all three distances. Differences among the trees occur mainly in the European and Jewish sections, with branching patterns that differ across trees for the four Jewish populations, for the Adygei population, and for the Sardinians and Tuscans.

**Figure 4:**
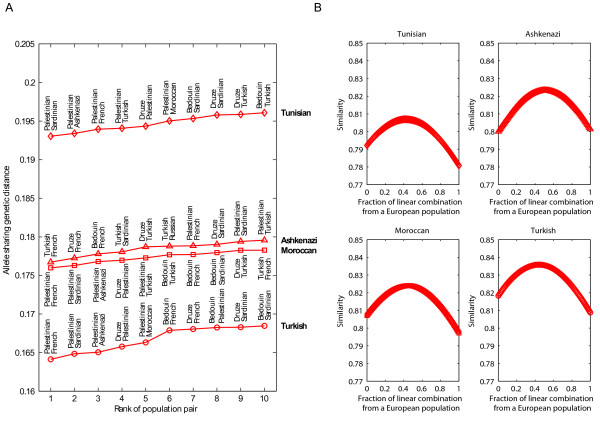
**Similarity to Jewish populations of linear combinations of pairs of populations**. (a) The highest-ranking population pairs for each Jewish population according to the minimal allele-sharing genetic distance between the Jewish population and the most similar linear combination of the population pair. The Y-axis indicates the genetic distance to a Jewish population of each pair, measured using the most similar linear combination for the pair. (b) Genetic similarity to a Jewish population (one minus genetic distance) of a linear combination of two populations, as a function of the coefficients in the linear combination. For each Jewish population, the plot shows only the population pair with the most similar linear combination to the Jewish population. The coefficients of the linear combination that produced the greatest similarity were 0.44 and 0.56 for French and Palestinians as the highest-ranking pair of the Turkish Jews (similarity 0.8359), 0.45 and 0.55 for French and Palestinians as the highest-ranking pair of the Moroccan Jews (similarity 0.8240), 0.50 and 0.50 for French and Turkish Jews as the highest-ranking pair of the Ashkenazi Jews (similarity 0.8233), and 0.42 and 0.58 for Sardinians and Palestinians as the highest-ranking pair of the Tunisian Jews (similarity 0.8070).

Because the results from clustering and population trees suggest similarity of Jewish populations to both European populations and Middle Eastern populations, we next examined whether allele frequencies in each of the Jewish populations could be described by linear combinations of the allele frequencies from pairs of other populations in the study. For each Jewish population, Figure 4A shows the ten highest-ranking population pairs according to the minimal allele-sharing genetic distance. For each pair of populations and each Jewish population, the coefficients in the linear combination were chosen such that the genetic distance between the linear combination and the Jewish population was minimized (Figure 4B). For example, the linear combination of populations with smallest genetic distance to the Turkish Jews consists of French (with a coefficient of λ = 0.44) and Palestinians (coefficient 1-λ = 0.56). French and Palestinians also provide the most similar pair for Moroccan Jews, with coefficients very nearly equal to the values in the case of Turkish Jews (λ = 0.45 for French). The most similar pair for Ashkenazi Jews consists of French and Turkish Jews (λ = 0.50), whereas for Tunisian Jews the most similar pair consists of Sardinians and Palestinians (λ = 0.42 for Sardinians). For all four Jewish populations, many of the ten closest pairs of populations consist of one Middle Eastern population and either one European population or one of the other Jewish populations. Additionally, because the Y-axis of Figure 4A indicates the distance of each Jewish population to combinations of other populations, the order of the four lines from top to bottom indicates the relative distinctiveness of the Jewish populations. Tunisian Jews are most genetically distinctive with respect to the other populations in the dataset, followed by Ashkenazi Jews, Moroccan Jews, and Turkish Jews.

**Figure 5:**
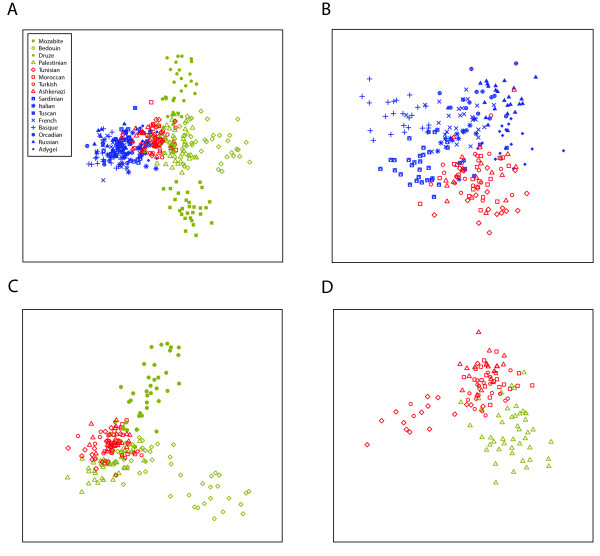
**Multidimensional scaling (MDS) analysis of population structure**. (a) MDS for European, Jewish, and Middle Eastern individuals. (b) MDS for European and Jewish individuals. (c) MDS for Jewish and Middle Eastern individuals, excluding Mozabites. (d) MDS for Palestinian and Jewish individuals.

As another method of investigating population structure at an individual level, we examined multidimensional scaling (MDS) representations of the distances between pairs of individuals (Figure 5A). The four Middle Eastern populations are placed in largely distinct locations in the MDS representation, whereas the various European and Jewish populations are placed in locations that overlap to a greater extent. The Jewish populations are located between the European and Middle Eastern populations, with the Ashkenazi Jewish individuals placed closer to the Europeans. This placement of the Ashkenazi population is reflected in an average linkage distance of *L*_0 _= 0.0451 between the Ashkenazi group and the pooled Europeans, compared with corresponding *L*_0 _values of 0.0533, 0.0560, and 0.0591 for the Turkish, Tunisian, and Moroccan Jewish populations, respectively. The probability is *P *< 0.001 that permutation of the Jewish population labels produces a lower average linkage distance between the permuted Ashkenazi population and the Europeans. By contrast, the corresponding *P*-values - based on the same permutations of the labels - are 0.500, 0.854, and 0.990 for the Turkish, Tunisian, and Moroccan Jewish populations, respectively.

To investigate the possibility of further separation of the European and Jewish individuals, we also examined the MDS representation of distances for these individuals alone (Figure 5B). The Tunisian Jews are located further from the pooled European populations than are any of the other Jewish populations, with *L*_0 _= 0.1180 and *P *> 0.999 based on permutation of the Jewish population labels, compared with *L*_0 _= 0.0859 (*P *= 0.005), *L*_0 _= 0.0899 (*P *= 0.065), and *L*_0 _= 0.0946 (*P *= 0.327) for the Turkish, Ashkenazi, and Moroccan Jewish populations, respectively. The European populations that cluster closest to the pooled Jewish populations are the Tuscan, Italian, Sardinian, and Adygei populations, each with *P *< 0.001 based on permutations of the European population labels.

In a similar way, we also examined the MDS representation for the Middle Eastern and Jewish populations alone, excluding the relatively distinctive Mozabites. As can be seen in Figure 5C, the Palestinians are relatively close to the pooled Jewish populations (*L*_0 _= 0.0488, *P *< 0.001 in permutations of the labels among the Bedouin, Druze, and Palestinian populations), whereas the Bedouin and Druze populations are more separated from the pooled Jewish populations (*L*_0 _= 0.1102, *P *> 0.999, and *L*_0 _= 0.1023, *P *= 0.990, respectively) and largely produce distinctive clusters of their own.

Because the closest population in Figure 5C to the four Jewish populations was the Palestinian population, we also considered the MDS representation of only the Jewish populations together with the Palestinians (Figure 5D). The plot places the Palestinians closer to the Moroccan and Turkish Jews than to the other Jewish populations (*L*_0 _= 0.1153, *P *= 0.024, and *L*_0 _= 0.1009, *P *< 0.001 for Moroccan and Turkish Jews, respectively, in permutations of the labels among the Jewish populations, in contrast with *L*_0 _= 0.1356, *P *= 0.847, and *L*_0 _= 0.1632, *P *> 0.999 for Ashkenazi and Tunisian Jews, respectively). It further suggests that the Tunisian Jews are the most distinctive Jewish population, whereas the Ashkenazi, Turkish, and Moroccan Jewish populations are genetically more similar to each other.

### Separate analysis of Jewish populations

**Figure 6:**
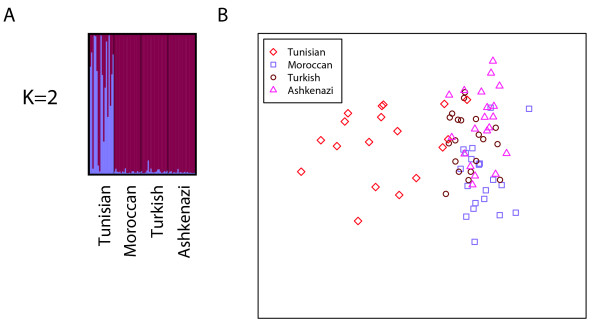
**Jewish population structure**. (a) Inference using unsupervised clustering. (b) Multidimensional scaling analysis for Jewish individuals. In the unsupervised clustering analysis, the predefined number of clusters was *K *= 2. The most frequent mode found by *Structure *for *K *= 2 included all 40 replicates. For higher values of *K*, only modes with small numbers of replicates were found (not shown).

Focusing on the Jewish populations alone, we again used *Structure *with an admixture model to cluster individuals in an unsupervised manner. Figure 6A shows the graphical representation of the clustering for *K *= 2, confirming the relative distinctiveness of the Tunisian Jews from the Ashkenazi Jews, Moroccan Jews, and Turkish Jews, which were not separated in this analysis. However, an MDS representation of the four Jewish populations shows that Moroccan Jews, Tunisian Jews, and Ashkenazi Jews can be largely separated (Figure 6B). The Turkish Jews are not easily distinguished from the Ashkenazi and Moroccan Jews in the MDS analysis, and are placed in positions overlapping with the Ashkenazi and Moroccan Jewish individuals.

## Conclusion

To examine the affinities of Jewish populations and their relationships to Middle Eastern and European populations, we have analyzed a sample of 78 individuals from four Jewish populations at 678 autosomal microsatellite loci together with corresponding genotypes of 321 Middle Eastern and European non-Jewish individuals from 12 populations. In various statistical analyses of population structure, the Jewish populations had a high level of genetic similarity to each other, grouping together in Bayesian clustering (Figure 2), neighbor-joining population trees based on three population-level genetic distances (Figure 3), and MDS analysis based on individual-level genetic distances (Figure 5). Moreover, in multiple analyses the Jewish populations were placed in an intermediate position in relation to the European and Middle Eastern populations, both in analyses of genetic variability (Table 1 and Figure 1) and in analyses of population structure (Figures 2, 3, and 5). When we searched for linear combinations of population pairs that produced minimal genetic distance to Jewish populations, the minima were often obtained from pairs that included one European population and one Middle Eastern population in similar proportions (Figure 4).

Whereas recent Y-chromosomal studies have identified a trend of genetic affinity among Jewish populations [12,15,18], most notably a shared group of haplotypes common in Jewish priests from different Jewish populations [16,52,53], past autosomal studies of multiple Jewish populations have been somewhat more equivocal regarding the clustering of Jewish populations separate from non-Jewish populations [13,14,17,19,54-56]. Recent genomic studies that have identified a component of distinctive ancestry for Jewish individuals have largely focused on Ashkenazi Jews sampled in the United States in relation to the broader European-American population [7-10], finding most recently that individuals with even partial Ashkenazi ancestry can be detected on the basis of principal components analysis [10]. Our study furthers the results of these studies by showing that a distinctive component of genomic ancestry extends to Jewish populations more broadly.

A simple explanation for the clustering of the Jewish populations is that this pattern is the consequence of shared ancestry with an ancestral Middle Eastern group. Under this scenario, the intermediate placement of the Jewish populations with respect to European and Middle Eastern populations would then result from early shared ancestry of the Jewish and Middle Eastern populations, followed by subsequent admixture of the Jewish populations that took place with European groups or other groups more similar to the Europeans than to the Middle Eastern populations in the study. Although it is difficult to assess the specific nature of the admixture on the basis of our analysis, this explanation is supported by other genetic studies that find a combination of shared ancestry and admixture among Jewish populations [56-59] and by historical records of conversions to Judaism [20,60-64]. Further sampling of matched Jewish and neighboring non-Jewish populations will be informative for investigating the evidence for this scenario.

One frequently discussed conversion that likely occurred in the 8th century at the far eastern edge of Europe, north of the Caucasus and Black Sea regions, is that of the Khazarian kingdom [60,62,64]. The demographic effect of this conversion is debated, so that only a small minority of the Khazars may have adopted Judaism. While the ultimate fate of the Khazar population remains unknown, the theory has been advanced that a large fraction of the ancestry of eastern European Jews derives from the Khazars [60,62-64]. This theory would predict ancestry for the eastern European Ashkenazi Jewish population to be distinct from that of the other Jewish populations in the study. Although we did not observe such a distinct ancestry, it is noteworthy that in some analyses (Figures 2 and 3), as was observed in the recent study of Need *et al*. [10], we did detect similarity of the Adygei, a north Caucasian group from the area once occupied by the Khazars, to the Jewish populations.

In several analyses, the population in the study that is most similar to the Jewish populations is the Palestinian population. This result is reflected by the fact that for *K *= 5, Bayesian clustering with *Structure *assigns the Jewish populations and the Palestinians to the same cluster (Figure 2), and by the relatively close placement of the Palestinians and the Jewish populations in MDS plots of individual distances (Figure 5). This genetic similarity, which is supported by several previous studies [12,65,66], is compatible with a similar Middle Eastern origin of the Jewish populations and the Palestinians. Admixture of the Palestinians with groups with European origins might have maintained or augmented this shared ancestry, especially if it was paralleled with similar admixture of these groups with Jewish populations.

Among the Jewish populations, the Tunisians were found to be the least variable and most distinctive, and their genotypes could be most easily distinguished from those of the three other Jewish populations. This result suggests a smaller population size and greater degree of genetic isolation for this population compared to the other Jewish groups, or a significant level of admixture with local populations. These explanations are not incompatible, as it is possible that early admixture was followed by a long period of isolation. Some Berber admixture of Tunisian Jews may very well have taken place [61,63], and documentation of rare Mendelian disorders in Tunisian Jews [67-69] supports a view of isolation with relatively few founding individuals. A smaller-scale autosomal study that did not include Tunisian Jews found the neighboring Libyan Jewish population to be distinctive with respect to other Jewish populations [66], and our results concerning the Tunisian Jewish population might reflect a similar phenomenon.

We note that caution is warranted in interpreting some of our results. For example, in the population trees produced from three distance measures (Figure 3) there is disagreement on the branching order of three of the European populations closest to the Jewish populations (Adygei, Sardinian, and Tuscan). Thus, from these data, it is difficult to make strong inferences regarding the most similar European populations to Jewish groups. However, consistent with studies that have incorporated a single Jewish population in a broader European context [6-9], southern groups from Europe are placed closer to the Jewish populations than more northerly groups. An additional disagreement among the trees lies in the branching pattern of the Jewish populations themselves. However, this within-group disagreement does not affect the basic pattern visible in all three trees, in which the Middle Eastern and European populations cluster separately with the Jewish populations in the center. A possible additional concern is ascertainment bias on the loci favoring high levels of European polymorphism. However, no strong evidence for ascertainment bias has been detected for the loci considered here [70], and in general, ascertainment effects in humans are only significant in studies of populations from distant geographic regions. Two recent genomic studies of Ashkenazi Jews sampled in the United States [10,71] have demonstrated the potential of the use of haplotypes and extremely densely placed markers for detailed investigation of genetic variation in a Jewish population, and it is possible that with the resolution provided by higher densities and haplotypic analysis, some of the discrepancies in our analyses might be overcome. Irrespective of the limitations of our study, however, our main results, namely the clustering of the Jewish populations and the intermediate placement of the Jewish populations compared to European and Middle Eastern populations, were robust across diverse types of analysis.

## Authors' contributions

Designed the study: MWF, JH, NAR; collected the samples: DG, JH; performed the data analysis: NMK, CW; supervised the data analysis: LS, MWF, JH, NAR. All authors contributed to writing the manuscript and provided their approval.
